# Effect of Heat Treatment on the Chemical Structure and Thermal Properties of Softwood-Derived Glycol Lignin

**DOI:** 10.3390/molecules25051167

**Published:** 2020-03-05

**Authors:** Thi Thi Nge, Yuki Tobimatsu, Masaomi Yamamura, Shiho Takahashi, Eri Takata, Toshiaki Umezawa, Tatsuhiko Yamada

**Affiliations:** 1 Center for Advanced Materials, Forestry and Forest Products Research Institute (FFPRI), 1 Matsunosato, Tsukuba, Ibaraki 305-8687, Japan; stakahas@affrc.go.jp (S.T.); takaeri@affrc.go.jp (E.T.); 2 Research Institute for Sustainable Humanosphere, Kyoto University, Gokasho, Uji, Kyoto 611-0011, Japan; ytobimatsu@rish.kyoto-u.ac.jp (Y.T.); yamamura@rish.kyoto-u.ac.jp (M.Y.); tumezawa@rish.kyoto-u.ac.jp (T.U.); 3 Research Unit for Development and Global Sustainability, Kyoto University, Gokasho, Uji, Kyoto 611-0011, Japan

**Keywords:** glycol lignin, polyethylene glycol (PEG) solvolysis, wood meal size, heat treatment, 2D HSQC NMR, thioacidolysis, thermal flow property

## Abstract

A large-scale glycol lignin (GL) production process (50 kg wood meal per batch) based on acid-catalyzed polyethylene glycol (PEG) solvolysis of Japanese cedar (JC) was developed at the Forestry and Forest Products Research Institute (FFPRI), Tsukuba, Japan. JC wood meal with various particle size distributions (JC-S < JC-M < JC-L) (average meal size, JC-S (0.4 mm) < JC-M (0.8 mm) < JC-L (1.6 mm)) and liquid PEG with various molecular masses are used as starting materials to produce PEG-modified lignin derivatives, namely, GLs, with various physicochemical and thermal properties. Because GLs are considered a potential feedstock for industrial applications, the effect of heat treatment on GL properties is an important issue for GL-based material production. In this study, GLs obtained from PEG400 solvolysis of JC-S, JC-M, and JC-L were subjected to heating in a constant-temperature drying oven at temperatures ranging from 100 to 220 °C for 1 h. All heat-treated GL series were thermally stable, as determined from the Klason lignin content, TMA, and TGA analyses. SEC analysis suggests the possibility of condensation among lignin fragments during heat treatment. ATR-FTIR spectroscopy, thioacidolysis, and 2D HSQC NMR demonstrated that a structural rearrangement occurs in the heat-treated GL400 samples, in which the content of α–PEG-*β*–*O*-4 linkages decreases along with the proportional enrichments of *β*–5 and *β*–*β* linkages, particularly at treatment temperatures above 160 °C.

## 1. Introduction

Lignin is known as the most abundant aromatic biopolymer in nature, representing 15%–35% of the cell wall components of typical vascular plants including trees, where it functions as a bonding agent between cells to provide cell walls with outstanding resistance against external physical, chemical, and biological reactions [1]. Lignin is readily available in large quantities as a major byproduct of pulp and paper plants and lignocellulosic biofuel industries. Lignins from chemical pulping processes are primarily used as fuel feedstock, while only a small portion of technical lignins in the form of kraft lignin, alkali lignin, and lignosulfonate are used for value-added products because of their diverse chemical structures and heterogeneity, which largely depend on the sources, processing conditions, and isolation method [2,3,4]. Lignins typically consist of three types of phenylpropane units, i.e., p-hydroxyphenyl (H), guaicyl (G), and syringyl (S) units, with various interunit linkages such as *β*–*O*–4 (40%–60%), *β*–5 (4%–10%), and biphenyl (3.5%–25%) [5]. Depending on the type of biomass resource used (e.g., softwood, hardwood, or grass), different proportions of phenylpropane units and diverse interunit linkages occur, resulting in wide variations in lignin macromolecules, with a high degree of complexity and heterogeneity [6,7,8]. Because of these heterogeneous features, high-value applications of technical lignins are rather limited. It is difficult to change the process conditions to obtain lignin with specific properties, as lignin is only a byproduct. Numerous studies and developments on effective isolation methods to obtain lignin with specific properties have been reported [9,10,11,12,13], and some of these approaches have been integrated in chemical pulping processes and realized in large-scale plants [9,10,11]. In addition, the organosolv fractionation method has been widely used to isolate lignin from biomass, for which efficient lignin isolation yields with high purity and high pulp yield have been reported [14].

Considering the issues of structural heterogeneity and the subsequent processing required for lignin byproducts, we propose the use of softwood as an initial biomass resource to produce lignin derivatives as the main product. Because softwoods are known to contain lignins almost exclusively composed of G units, lignin derivatives can be produced with less structural heterogeneity in terms of the aromatic unit [1,15]. Utilizing the abundance of Japan’s softwood plantation forests, 50% of which are occupied by Japanese cedar (*Cryptomeria japonica;* JC), we have developed a large-scale (50 kg wood meal per batch) lignin production process based on technically feasible and environmentally benign acid-catalyzed polyethylene glycol (PEG) solvolysis of JC. A test plant was developed in 2015 at the Forestry and Forest Products Research Institute (FFPRI), Tsukuba, Japan, under a project of the Strategic Innovation Promotion (SIP) Lignin Research Consortium [16]. JC wood meals with various particle size distributions (JC-S < JC-M < JC-L) and liquid PEG with various molecular masses (PEG200 < PEG400 < PEG600) are typically used as starting raw materials to produce PEG-modified lignin, namely, glycol lignin (GL). The effectiveness of the production process has been elucidated by investigating the influence of these two main raw materials on the yield, chemical structure, average molecular mass, and thermal properties of the resulting GLs when operating under constant reaction conditions. It has been found that the chemical structure of GLs contains three main intermonomeric linkage types, i.e., α-PEG-*β*–O–4, *β*–5, and *β*–*β* linkages, indicating that the substitution of PEG moieties occurs primarily at the benzyl (α) carbon in α-OH-*β*–O–4 units in the source JC lignins during PEG solvolysis, giving rise to α-PEG-*β*–O–4 units. The PEGylation of lignin exhibits a viscous thermal flow at temperature above the glass transition state, providing suitable space for the molecular motion of rigid lignin side chains [16,17]. This type of thermal fusibility is crucial for thermal processing in material fabrication. Hence, the application of GLs to value-added functional materials, including flexible electronic substrates [18,19], fiber-reinforced plastics [20], elastomeric epoxy resins [21], and heat-resistant insulation films [22], has been explored, aiming to promote large-scale GL utilization in various applications, particularly for high-value-added GL-based high-performance materials.

Because GLs are considered a potential feedstock for industrial applications, the effect of heat treatment on GL properties is an important parameter to consider for efficiently integrating GL with other counterparts and to obtain the intended characteristics of the target products. A continuous supply with constant qualities as a common feedstock is another goal for potential industrial implementations of GL-based material production. It is well known that heat treatment alters the chemical structure of lignin as well as its molecular weight due to thermal decomposition and structural rearrangement through a series of chemical reactions, depending on the treatment temperature and duration [23,24,25,26]. In this study, three types of PEG400-substituted GL (GL400) samples obtained from the test plant were subjected to heat treatment in a constant-temperature drying oven at temperatures ranging from 100 to 220 °C for 1 h. To obtain an in-depth understanding of the physicochemical properties of heat-treated GL400 samples, ultraviolet (UV) and Klason lignin analyses, size exclusion chromatography (SEC), attenuated total reflectance Fourier-transform infrared (ATR-FTIR) spectroscopy, thioacidolysis, 2D nuclear magnetic resonance (NMR), thermomechanical analysis (TMA), and thermogravimetric analysis (TGA) were performed to determine the lignin content, average molecular mass, functional group distribution, frequency of ether linkages and intermonomeric linkages, and thermal transition temperatures.

## 2. Results and Discussion

**Scheme 1:**
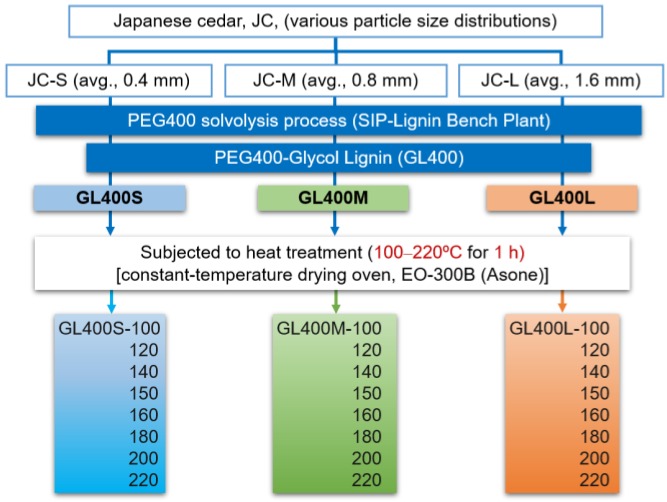
Schematic illustration of heat treatment for three types of PEG400-modified glycol lignin (GL400). GL400S, GL400M, and GL400L were obtained from the SIP-Lignin Bench Plant through acid-catalyzed PEG400 solvolysis of wood meal (JC): JC-S, JC-M, and JC-L, respectively.

As reported in our previous study, the particle size distribution of the source wood meal (JC) strongly affects the molecular mass, chemical structure, and thermal properties of the resultant GLs. For example, a greater source wood meal size (an average size of 0.4–1.6 mm was applied in the present study range) gives rise to GLs with a higher average molecular mass, higher yield of *β*–*O*–4-linked units, greater degree of PEGylation, and slightly reduced thermal transition temperatures, such as glass transition temperature (*T*_g_), viscous thermal flow temperature (*T*_f_), decomposition starting temperature (*T*_dst_), and maximum degradation temperature (*T*_dmax_) [16]. Hence, three types of control GL samples, denoted as GL400S, GL400M, and GL400L (Table 1), produced from PEG400 solvolysis of JC-S, JC-M, and JC-L, respectively, were selected for thermal treatment. As shown in Scheme 1, the GL400 samples were subjected to heat treatment at temperatures ranging from 100 to 220 °C in a constant-temperature drying oven for 1 h under atmospheric conditions. The heat-treated GL400 samples were designated based on the GL400-source wood meal size and treatment temperature, e.g., GL400S-100, GL400M-100, GL400L-100, etc. (Scheme 1). The visual color change from pale brown to dark brown observed with increasing temperature suggests that thermochemical reactions, such as depolymerization, side chain cleavage, and repolymerization through condensation, may occur during heat treatment [23,24,25,26], even though only a trace amount of weight loss (less than 1 wt%) was measured after heat treatment.

### 2.1. Lignin Content of Heat-Treated GL400

**Figure 1:**
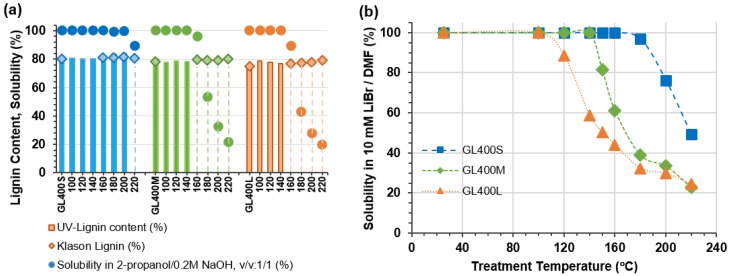
Lignin content (UV and Klason) of GL400S, GL400M, and GL400L after heat treatment at temperatures of 100 to 220 °C for 1 h and their solubility in the 2-propanol:0.2M NaOH (*v*/*v*:1/1) solvent used for UV lignin analysis (**a**) and in the 10 mM LiBr/DMF solvent used for SEC analysis (**b**).

The lignin content of the heat-treated GL400 series was determined by either UV or Klason lignin analysis, as not all samples completely dissolved in the solvent (2-propanol:0.2M NaOH, *v*/*v*: 1/1) used for UV analysis, hereafter denoted as the UV solvent. In particular, low solubilities were observed for GL400M and GL400L samples treated at temperatures above 150 °C. The insoluble portion of the GL400M and GL400L series increased with increasing treatment temperature, whereas the GL400S series remained nearly soluble at a treatment temperature of 200 °C. The UV lignin contents of completely soluble samples were found to be similar regardless of the treatment temperature (Figure 1a). In addition, the Klason lignin contents determined for samples treated at 160, 180, 200 and 220 °C for each GL400 series were also similar, although a slight increase was observed with increasing temperature compared with the corresponding GL400 controls (Figure 1a, Table 1). The results of UV and Klason lignin analyses suggest that no significant degradation occurs at temperatures of up to 220 °C for the three GL400 series [24]. However, it appears that a heat-induced structural rearrangement occurs in the GL400M and GL400L series at treatment temperatures above 150 °C, which generate a more condensed structure, as indicated by the UV-solvent insoluble fractions. The UV-solvent soluble fractions most likely consist of GL400 fractions with a higher degree of PEGylation compared with the UV-solvent insoluble fractions, while the PEG content of the heat-treated samples remains largely unchanged.

### 2.2. Molecular Weight Distribution of Heat-Treated GL400

The average molecular mass (*M*_w_ and *M*_n_) of the control GL400S, GL400M, and GL400L samples subjected to heat treatment are 5310/2050, 8150/2360, and 13,630/2590, respectively (Table 1). Low solubility was also observed for the heat-treated GL400 series in the eluent (10 mM LiBr/DMF) used for SEC analysis, which determines the average molecular mass. Low solubilities were observed in samples treated at ≥180 °C in the GL400S series, ≥ 150 °C in the GL400M series, and ≥ 120 °C in the GL400L series (Figure 1b). The variation in low solubility with GL400 type at different treatment temperatures implies that the source wood meal size for the GL400 has a substantial effect.

The average molecular mass (*M*_w_ and *M*_n_) increased with increasing temperature in all completely soluble heat-treated samples, and the increase in *M*_w_ values was greater than that of the *M*_n_ values (Table 1, Figure S1). For a treatment temperature of 220 °C, a considerably higher molecular mass (*M*_w_, 7060) was determined for the moderately soluble fraction of GL400S (49% solubility), whereas lower *M*_w_ values of 2110 and 1840 were determined for the partially soluble fractions of GL400M (24% solubility) and GL400L (24% solubility), respectively, compared with the corresponding control samples (Table 1). SEC data revealed that the DMF-insoluble fractions of heat-treated samples in each GL400 series were modified GL400 fractions (heat-induced structural rearrangements, such as a cross-linked and condensed structure) with a considerably higher molecular mass. The extent of this modification was related to the treatment temperature [23]. The DMF-soluble fractions, however, were low-molecular-mass GL400 fractions having PEG–lignin side chains that were not affected by the heat-induced structural rearrangement.

### 2.3. Chemical Structure of Heat-Treated GL400 Samples

**Figure 2:**
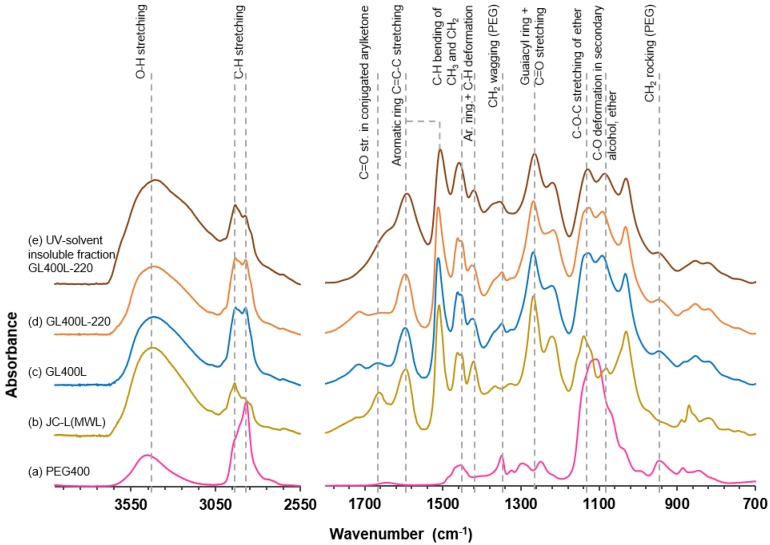
ATR-FTIR spectra of PEG400 (**a**), milled wood lignin (MWL) extracted from JC wood meal (JC-L) (**b**), GL400L prepared from acid-catalyzed PEG400 solvolysis of wood meal (JC-L) (**c**), GL400L after heat treatment at 220 °C for 1 h, (GL400L-220) (**d**), and the fraction of heat-treated GL400L-220 that is insoluble in the solvent (2-propanol:0.2M NaOH, *v*/*v*:1/1) used for UV lignin analysis (**e**).

ATR-FTIR spectra of PEG400 (a), milled wood lignin (MWL) extracted from JC-L (b), control GL400L (c), GL400L treated at 220 °C, termed GL400L-220 (d), and the UV-solvent insoluble fraction of GL400L-220 (e) are displayed in Figure 2. The absorption peaks observed at 2870, 1350, 1103–1106, and 947 cm^−1^ for PEG400 (Figure 2a) correspond to symmetric C–H stretching of methylene groups, CH_2_ wagging, and C–O stretching, and CH_2_ rocking vibration [27,28], with strong C–O stretching absorption proximal to 1103 cm^−1^ being the dominant contribution. Consequently, the appearance of two strong peaks at 1126 cm^−1^ (C–O–C stretching of ether) and 1092 cm^−1^ (C–O deformation in secondary alcohol and ether) in GL400L (Figure 2c), compared with the MWL spectrum (Figure 2b) indicate the introduction of PEG moieties into the lignin macromolecules. In addition, two new absorption peaks at 1350 and 947 cm^−1^ and the enhanced intensity of the C–H stretching vibration at 2873 cm^−1^ and of the characteristic lignin absorption peaks in the GL400 spectrum further indicate the PEGylation of lignin macromolecules.

The typical absorption peaks of lignin [29] (Figure 2c) are assigned to aromatic and aliphatic O–H stretching (ca. 3410-3413 cm^−1^), asymmetric C–H stretching of methyl and methylene groups (2933 cm^−1^), unconjugated (ca. 1714 cm^−1^) and conjugated (ca. 1666 cm^−1^) carbonyl stretching in ketone/aldehyde groups, aromatic skeletal vibration or aromatic ring C=C–C stretching (1597 and 1512 cm^−1^), C–H deformation of methyl and methylene groups (1462-1454 cm^−1^), aromatic skeletal vibration combined with C–H deformation (1423-1427 cm^−1^), guaiacyl (G) ring breathing with C–O stretching (1269 cm^−1^), C–C, C–O, and C=O stretching of G ring (1221-1223 cm^−1^), and G-type aromatic C–H in-plane deformation and C–O deformation (1034 cm^−1^). The ATR-FTIR spectrum of GL400L after heat treatment at 220 °C (Figure 2d) displays common features in several regions compared with that of the corresponding control GL400L, whereas a notable difference is observed in the spectrum of its UV-solvent insoluble fraction (Figure 2e). The O–H absorption peak centered at 3417 cm^−1^ for GL400L-220 (Figure 2d) was shifted to a lower wavenumber of 3410 cm^−1^, with a broad peak for the UV-solvent insoluble GL400L-220. In addition, a notably enhanced intensity at 1653 cm^−1^ (carbonyl stretching in conjugated aryl ketone) and a weakened unconjugated carbonyl absorption peak at 1714 cm^−1^ were observed in the UV-solvent insoluble fraction (Figure 2e). It is possible that the cleavage of *β*–*O*–4 linkages under heat treatment involves the formation of ketones via reactive benzyl cations [7,23,30].

A semiquantitative examination was performed to measure the relative absorbance using the peak at 1512 cm^−1^ (aromatic ring C=C–C stretching) as an internal reference to normalize all spectra. The measured relative intensities of selected peaks for the GL400L and GL400M series treated at 200–220 °C and their respective UV-solvent insoluble fractions are listed in Table 2 and Table 3, respectively.

As displayed in Table 2 for the GL400L series, the reduced relative intensity at 949–951 cm^−1^ and the increased relative intensity at 1354–1356 cm^−1^ for PEG in the UV-solvent insoluble fractions indicate that the PEG–lignin side chains are involved in the structural rearrangement. Moreover, compared with the corresponding counterparts, the increase in relative intensity at 1421 and 1460 cm^−1^ for the UV-solvent insoluble fraction, corresponding to aromatic skeletal vibration combined with C–H in-plane deformation and C–H bending of CH_3_ and CH_2_ groups, suggests an enrichment in condensed bonds with increasing treatment temperature. In addition to the increased intensity, the broad absorption peak at 1593 cm^−1^ (aromatic skeletal vibration combined with C=O stretching) further supports the presence of structural diversity around the aromatic rings. The proportional increase in aromatic C–C bonds, such as *β*–5 and *β*–*β*, mainly due to the decomposition of *β*–*O*–4 ether linkages, is also supported by the HSQC NMR data discussed below. The same trend was observed for the heat-treated GL400M series (Table 3). Overall, the FTIR spectral data provide evidence of structural rearrangements occurring during heat treatment, particularly at ≥ 180 °C, where the UV-solvent insoluble portions were determined to account for 50%–80% depending on the GL400 type and treatment temperature (Figure 1a).

**Figure 3:**
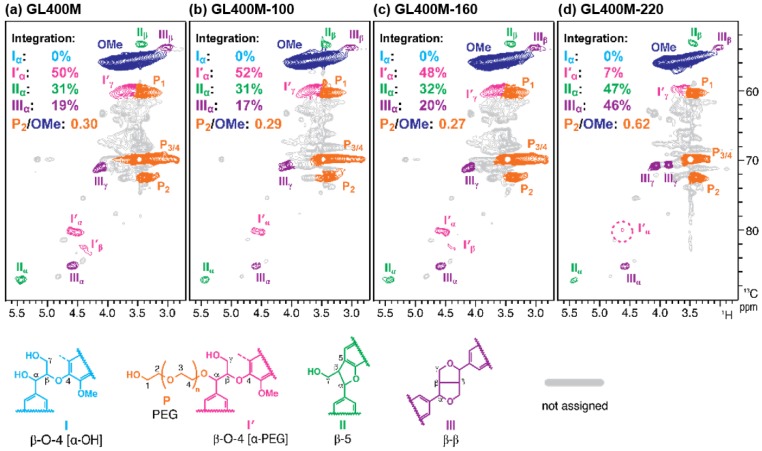
Two-dimensional (2D) HSQC NMR spectra of GL400M before heat treatment (**a**) and after heat treatment at 100 °C (**b**), 160 °C (**c**), and 220 °C (**d**) for 1 h. GL400M was obtained via acid-catalyzed PEG400 solvolysis of JC wood meal (JC-M).

For a further in-depth structural characterization of heat-treated GL400 samples, thioacidolysis and 2D HSQC NMR (Table 1 and Figure 3) were performed. The cleavage of *β*–*O*–4 linkages is a common feature of many lignin depolymerization and degradation pathways for any type of thermal treatment [23,24,30]. Supporting this contention, analytical thioacidolysis, which quantifies lignin monomers released from *β*–*O*–4 units upon chemical degradation [31,32], of the heat-treated GL400 samples indicated that the amount of *β*–*O*–4 units per Klason lignin (Table 1) decreased with different degrees depending on the treatment temperature and the size of the source wood meal. In consistent with our previous work [16], the monomer yield of control GL400 samples decreased with decreasing source wood meal size (GL400L > GL400M > GL400S). For the same treatment temperature range of 100–220 °C, the GL400S series showed little or relatively minor change, whereas the GL400M and GL400L series showed a sharp decrease in the thioacidolysis monomer yield at treatment temperatures of ≥ 200° and ≥ 160 °C, respectively (Table 1). Thioacidolysis data therefore suggest that the source wood meal size used for GL production affect not only the amount of *β*–*O*–4 units in GL400 [16] but also their chemical rearrangements upon the heat treatment.

Solution-state HSQC NMR spectra of selected heat-treated GL samples (Table 1) were recorded using DMSO-*d*_6_ as solvent; the solubility of each heat-treated GL sample was more or less similar to that in the LiBr/DMF system described earlier (Figure 1b). In consistent with our earlier work [16], the HSQC spectra of all the tested GL400 samples displayed signals from the major intermonomeric units, i.e., *α*–PEG-*β*–O–4 (**I’**), *β*–5 (**II**) and *β*–*β* (**III**), along with those from aromatic methoxyl (**OMe**) and PEG (**P**) residues, comprising lignin and PEG fractions for GL400 samples (Figure 3); signals from *α*–OH-*β*–O–4 (**I**) units abundant in the source JC lignin were undetectable in all the GL400 spectra (Figure 3), indicating their complete conversions during the solvolysis process to produce each GL sample. Relative intensities of well-resolved C_α_–H_α_ contours from *α*–PEG-*β*–O–4 (**I’**), *β*–5 (**II**) and *β*–*β* (**III**) units were used to estimate the change of lignin structures upon the heat treatment. Consequently, the proportions of *α*–PEG-*β*–O–4 (**I´**) were significantly depleted, whereas the proportions of *β*–5 (**II**) and *β*–*β* (**III**) units were instead increased, particularly at treatment temperatures above 160 °C (Figure 3, Table 1). The NMR data suggest that the chemical structure rearrangements primarily involve *β*–O–4 linkages, leaving *β*–5 and *β*–*β* linkages relatively unchanged during heat treatment at temperatures above 160 °C. We also observed that **P_2_**/**OMe** signal ratio, which reflects PEG/lignin ratio, was considerably increased in the spectra of GL400 samples treated at 220 °C compared with those observed in the spectra of the untreated GL400 samples and GL400 samples treated at 100 and 160 °C (Table 1, Figure 3). Given that large portions of GL400 samples treated at 220 °C became insoluble and inaccessible by NMR (Figure 1b), we deduced that relatively PEG-rich GL400 fractions left soluble (and accessible by NMR) after lignin condensation upon the heat treatment [23,25,30] had made relatively lignin-rich GL400 fractions insoluble (and inaccessible by NMR). This notion was corroborated by the SEC and ATR-FTIR data described earlier (Table 1, Table 2 and Table 3).

### 2.4. Thermal Properties of Heat-Treated GL400 Samples

TMA and TGA were performed to elucidate the thermal behavior of the heat-treated GL400 series. TMA measures the glass transition temperature (*T*_g_) and the viscous thermal flow temperature (*T*_f_), while TGA measures the decomposition starting temperature (*T*_dst_) and the maximum degradation temperature (*T*_dmax_).

**Figure 4:**
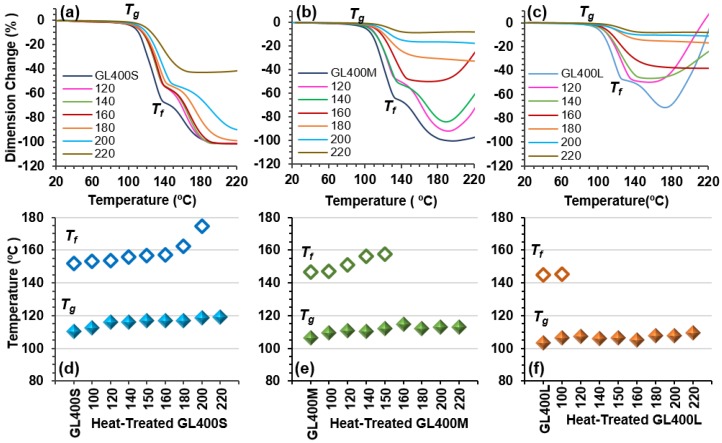
TMA profiles of the heat-treated GL400S (**a**), GL400M (**b**), GL400L series (**c**) and the respective thermal transition temperature, glass transition temperature (*T*_g_), and viscous thermal flow temperature (*T*_f_) for the GL400S (**d**), GL400M (**e**), and GL400L series (**f**). The samples were subjected to heat treatment at temperatures of 100–220 °C for 1 h.

As reported in our previous studies [16,17], the PEGylation of the lignin macromolecules gave rise to a second thermal transition temperature (*T*_f_) above *T*_g_ for all of the GLs, where the temperature range depends on the source wood meal size and the PEG molecular mass used for GL production. As the GLs in this study were produced from PEG400 solvolysis with three source wood meal sizes (JC-S < JC-M < JC-L), the corresponding GL400S, GL400M, and GL400L samples had *T*_g_ and *T*_f_ values of 110, 106, 104 °C and 152, 146, 145 °C, respectively. The GL400S produced from the smaller source wood meal had higher *T*_g_ and *T*_f_ values, even though its average molecular mass was lower than that of the other two series (Table 1). The *T*_g_ and *T*_f_ values are related to the degree of PEGylation and the relative proportions of major intermonomeric linkage units: *α*–PEG-*β*–O–4, *β*–5, and *β*–*β* (Table 1). Consequently, the lower proportions of *α*–PEG-*β*–O–4 signals and the higher proportions of *β*–*β* signals in GL400S compared with those of GL400M and GL400L give rise to higher *T*_g_ and *T*_f_ values. Both of the thermal transition temperatures for the heat-treated GL400S series gradually increased with increasing temperature up to 200 °C, where the increment in *T*_f_ was higher than that of *T*_g_, and *T*_f_ was not detected after heat treatment at 220 °C (Figure 4a,d). Notably, *T*_f_ was no longer detected in the GL400M (Figure 4b,e) and GL400L (Figure 4c,f) series after heat treatment at 160 and 100 °C, respectively. As stated above, the average molecular mass of the heat-treated GL400 series increased with increasing temperature, along with chemical structure rearrangements, including the enrichment of a cross-linked and condensed structure; the extent of this enrichment depended on the GL400 type. The molecular motion of the PEG–lignin side chains became limited, and *T*_f_ was no longer observed once the side chains movement was restricted.

**Figure 5:**
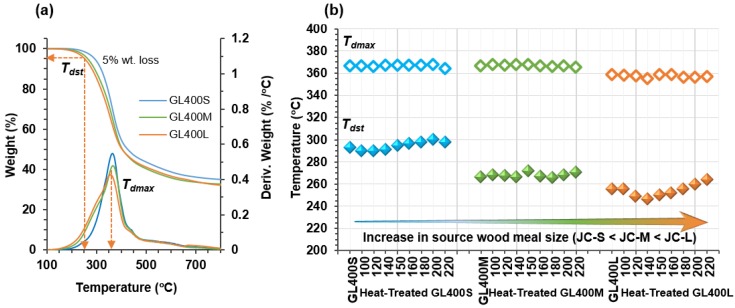
TGA/DTGA plots of control GL400S, GL400M, and GL400L (**a**) and thermal transition temperatures (*T*_dst_ and *T*_dmax_) for the heat-treated GL400S, GL400M, and GL400L series, as determined by TGA (**b**). The samples were subjected to heat treatment at temperatures of 100–220 °C for 1 h. *T*_dst_: decomposition starting temperature corresponding to 5 wt% loss, *T*_dmax_: temperature corresponding to the maximum weight loss.

In addition to *T*_g_ and *T*_f_, the thermal stability (*T*_dst_ and *T*_dmax_) of GLs is important for industrial utilization with consistent processability [33]. Generally, all of the heat-treated GL400 samples and corresponding control GL400 samples showed a broad DTGA curve with a maximum at 367 °C and shoulders near 300 and 430 °C; here, the shoulder near 300 °C is not well resolved in the GL400S series (Figure 5a), suggesting that GL400S series has a more condensed structure than Gl400M and GL400L series. *T*_dst_ (corresponding to 5 wt% weight loss) decreased slightly for treatment temperatures of up to 140 °C, followed by a gradual increase with increasing temperature. In contrast, *T*_dmax_ (corresponding to the maximum weight loss) showed no obvious change within the treatment temperature range for any of the GL400 series (Figure 5b).

The slight fluctuation of *T*_dst_ around 140 °C is related to the viscous thermal flow temperature (*T*_f_) of GL400, determined for 140–150 °C. An enhanced thermal stability was observed in all heat-treated GL400 samples after heating at temperatures above 140 °C. Both *T*_dst_ and *T*_dmax_ showed an increasing tendency with decreasing source wood meal size (GL400L series < GL400M series < GL400S series), as reported in our previous study [16]. The ranges of the TGA-derived *T*_dst_ for the GL400L, GL400M, and GL400S series were 246–264 °C, 267–272 °C, and 290–300 °C, respectively. The ranges of *T*_dmax_ were 356–359 °C, 364–367 °C, and 365–367 °C for the GL400L, GL400M, and GL400S series, respectively. In addition, the residual char at 800 °C increased slightly with increasing temperature for all GL400 samples: from 34.6 to 36.1 wt% (GL400S series), 30.5 to 33.6 wt% (GL400M series), and 31.9 to 33.3 wt% (GL400L series). This finding further indicates the enrichment of condensed structures with increasing treatment temperature.

In summary, three types of PEG400-substituted softwood-derived lignin derivatives, i.e., GL400S, GL400M, and GL400L, with unique physicochemical and thermal behaviors were subjected to heat treatment in a constant-temperature oven at temperatures of 100–220 °C for 1 h under atmospheric conditions. Klason lignin analysis indicated no obvious degradation in any of the GL400 series at temperatures of up to 220 °C. Instead, enhanced thermal stability was achieved after heating at temperatures above 140 °C for all GL400 samples, as supported by our *T*_dst_ results. This enhanced thermal stability is attributed to structural rearrangements occurring during heat treatment, particularly after heating at temperatures above 160 °C, as suggested by the thioacidolysis-derived *β*–*O*–4-linked monomer yields and HSQC NMR spectral data. It is likely that the cleavage of *β*–*O*–4-linked units (depolymerization) occurred simultaneously with a condensation reaction and that the repolymerization of lignin in a cross-linked condensed structure resulted in a considerable higher-molecular-mass fraction. Hence, a low solubility was observed for the heat-treated GL400 samples in multiple characterizing solvents used for UV lignin analysis, SEC analysis, and NMR analysis; however, the degree of solubility varied depending on the GL400 type and treatment temperature. ATR-FTIR spectral analysis of the UV-solvent insoluble Gl400L and GL400M fractions also provided evidence of chemical structural rearrangements during heating at higher temperatures. Overall, the heat-treated GL400 samples consist of PEG-rich soluble fractions with a relatively low average molecular mass and considerable high-molecular-mass fractions with a modified lignin structure (cross-linked condensed structure), which does not dissolve in characterizing solvents. However, the overall PEG content is not affected by moderate heat treatment. These results provide indispensable information for the advancement of GL production test plants to pilot-scale plants and for the upgrading of GLs with a wide variety of physicochemical and thermal properties to meet various industrial requirements for a potential feedstock.

## 3. Materials and Methods

### 3.1. GL Preparation and Heat Treatment

GL samples were obtained from the GL production test plant at FFPRI, Tsukuba, Japan [16]. Briefly, air-dried JC wood meal (46 kg (dry basis) per batch) with various particle size distributions (average wood meal size, JC-S (0.4 mm), JC-M (0.8 mm), JC-L (1.6 mm)), obtained as the byproducts of local sawmill industries, were used as received. The moisture content of JC wood meal is in the range of 9%–13%. PEG400 and 0.3 wt% sulfuric acid based on PEG were used as the solvolysis reagent and catalyst, respectively. Acid-catalyzed PEG solvolysis of JC (JC/PEG:1/5, *wt*/*wt*) was conducted in a large-scale batch reactor at 140 °C for 90 min under atmospheric pressure. The PEG400-modified GL (GL400) was collected as solid fractions after the acidification of lignin-solubilized solvolysis liquor and successive centrifugal washing (at 14,000 rpm for 30 min). Finely ground, vacuum- dried GL400 samples, denoted as GL400S, GL400M, and GL400L produced from JC-S, JC-M, and JC-L, respectively, were subjected to heat treatment.

A series of GL400 samples (ca. 10.00 g with moisture contents of less than 0.9%) were uniformly loaded in a glass petri dish (ϕ 60 mm, Pyrex^TM^) and subjected to thermal treatment in a constant-temperature drying oven (EO-300B, As One Corporation, Osaka, Japan) at designated temperatures ranging from 100 to 220 °C. The samples were placed in an oven that had been preheated to the designated temperature and maintained at the set temperature for 20 min. The heat treatment was then conducted for 1 h. All heat-treated samples were finely ground before centrifugal washing and were vacuum-dried for further analysis.

### 3.2. Chemical Analyses

UV lignin analysis was conducted with a Shimadzu UV1800 spectrophotometer (Shimadzu Corp., Kyoto, Japan). The heat-treated GL400 samples and corresponding control GL400 samples were typically dissolved in 2-propanol:0.2 M NaOH (*v*/*v*:1/1) solution followed by the addition of NaBH_4_. The solution samples were stored in the dark to allow the reduction reaction to proceed for 2 weeks in order to eliminate possible UV-absorbing furans. The solution was then diluted with an appropriate volume of 2-propanol:distilled water (*v*/*v*:1/1) to obtain UV absorption spectra within an intensity range of 0.3–0.7 at 280 nm. The lignin content was calculated using absorption coefficient values of 23.116, 21.563, and 20.955 gL^−1^cm^−1^ for the GL400S, GL400M, and GL400L series, respectively. Depending on the type of control GL400 sample, samples treated at >150 °C became completely insoluble in 2-propanol:0.2 M NaOH solution. The solubility of the heat-treated GL400 samples was also determined. The insoluble portion was filtered by vacuum-filtration, followed by successive washing before subjected to vacuum-drying. The percent solubility was then calculated based on an initial amount of sample used for corresponding analysis. Klason lignin analysis [16,34] was performed for samples hated at 160, 180, 200, 220 °C and for the corresponding control samples. Analytical thioacidolysis was performed to quantify *β*–*O*–4-linked lignin units in the GL400 samples, as reported previously [31,35,36]. Duplicate or triplicate runs were performed for each of the above chemical analyses, and the average values were reported.

### 3.3. D NMR

Samples heated at 100, 160, and 220 °C and corresponding control samples (ca. 15 mg) were dissolved in DMSO-*d*_6_ (600 μL) and filtered to remove the insoluble fraction. A similar solubility issue was encountered in samples treated at 220 °C. NMR spectra were recorded on a Bruker Biospin Avance III 800US spectrometer (Bruker Biospin, Billerica, MA, USA.) fitted with a cryogenically cooled 5 mm TCI gradient probe. Adiabatic 2D ^1^H–^13^C short-range correlation (HSQC) experiments were performed using the standard Bruker implementation (“hsqcetgpsp.3”, Bruker Biospin, Billerica, MA, USA), based on the parameters reported by Mansfield et al. [37]. The Bruker TopSpin software (Bruker Biospin, Billerica, MA, USA) was used for data processing, and the central DMSO solvent peaks (*δ*_C_/*δ*_H_: 39.5/2.49 ppm) were used as an internal reference. HSQC plots were obtained with typical matched Gaussian apodization in F2 and squared cosine-bell apodization and one level of linear prediction (16 coefficients) in F1. For contour integration analysis, well-resolved C_α_–H_α_ contours from **I**, **II**, **III**, and **I′**, and **OMe** and **P_2_** contours were integrated [16].

### 3.4. ATR-FTIR Spectroscopy

ATR-FTIR spectra were recorded using a Nicolet iS50 FTIR spectrometer (Thermo Fisher Scientific, Madison, WI, USA) equipped with an ATR accessary, a single crystal diamond top plate. Finely ground, vacuum-dried samples were used for spectrum collection over the range of 4000–400 cm^−1^ with an accumulation of 32 scans and a resolution of 4 cm^−1^. The band intensity of aromatic skeletal vibrations (aromatic ring C=C–C stretching) at 1512 cm^−1^ was used as an internal reference to normalize all spectra after ATR correction and base-line adjustment.

### 3.5. SEC

SEC analysis was performed using a Shimadzu Prominence LC-20AD (Shimadzu Corporation, Kyoto, Japan) system equipped with a UV (280 nm) and refractive index (RI) detector and a three-column sequence of Shodex KD802, KD803, and KD805. HPLC-grade *N*, *N*-dimethyl formamide (DMF) with 10 mM LiBr was used as the eluent, with a flow rate of 1.0 mL min^−1^ at 40 °C. Fluka polyethylene glycol/poly(ethylene oxide) standard ReadyCal sets (Sigma-Aldrich Japan G. K., Tokyo, Japan) were used for molecular mass calibration. The standard and samples were completely dissolved in the eluent and filtered through a 0.45 μm PTFE syringe filter (ADVENTEC) prior to injection to the system. Some of the GL400 samples treated at ≥120 °C did not dissolve completely, thus, the soluble portion was filtered and subjected to SEC analysis.

### 3.6. TMA

The glass transition temperature (*T*_g_) and the viscous thermal flow temperature (*T*_f_) of heat-treated GL400 samples and corresponding control samples were determined under a nitrogen environment (100 mL min^−1^) using a Q400 TMA (TA Instruments-Waters LLC, New Castle, DE, USA). A vacuum-dried, finely ground powder sample (7–8 mg) was loaded onto a platinum sample pan (ϕ 6 × 2.5 mm) and a flat aluminum plate (ϕ 4 mm) was placed on top of the sample to avoid direct contact with the probe surface. The sample assembly was placed between a quartz stage equipped with a thermal sensor and a movable probe with a contact diameter of 2.54 mm. The temperature was increased from 20 to 240 °C at a heating rate of 5 °C min^−1^ under an applied load of 0.05 N. Volume changes occurring within the samples during heating were recorded based on the motion of the probe. Universal analysis software was used to analyze the data, with *T*_g_ and *T*_f_ identified as onset temperatures.

### 3.7. TGA

The thermal stability of the heat-treated GL400 samples and corresponding control samples was determined under a nitrogen environment (60 mL min^−1^ for the sample chamber and 40 mL min^−1^ for the balance chamber) using a Q500 TGA (TA Instruments-Waters LLC, New Castle, DE, USA.). A vacuum-dried fine powder sample (7–8 mg) was loaded onto a TGA platinum pan (100 μL). The temperature was initially increased to 105 °C at 10 °C min^−1^ and was held constant for 20 min, followed by heating to 850 °C at a rate of 10 °C min^−1^. Universal analysis software was used to analyze the decomposition starting temperature (*T*_dst_) corresponding to 5 wt% loss and the maximum decomposition temperature (*T*_dmax_) corresponding to the maximum weight loss. *T*_dst_ was recalculated after the weight was fixed at 105 °C.

## Figures and Tables

**Table 1:** Chemical structure and molecular mass data for heat-treated GL400 Samples.

		**Thioacidolysis Monomer Yield (μmol/g) *^b^***		**Relative NMR Signal intensity and Ratio *^c^***				**Molecular Mass *^d^***	
**GL Samples *^a^***	**Klason Lignin (wt%)**	**per GL**	**per Klason Lignin**	**I** **’ [*β*–O–4] (%)**	**II** **[*β*–5] (%)**	**III** **[*β*–*β*] (%)**	**P_2_/OMe** **Ratio**	***M*_w_**	***M*_n_**
GL400S	79.8	14.7	18.4	32	20	49	0.30	5310	2050
GL400S-100	-	15.6	-	35	22	43	0.30	5450	2060
GL400S-120	-	15.2	-	-	-	-	-	6150	2130
GL400S-140	-	15.9	-	-	-	-	-	6910	2150
GL400S-150	-	15.8	-	-	-	-	-	7650	2220
GL400S-160	80.7	13.8	17.0	23	24	52	0.29	9220	2280
GL400S-180	80.8	16.2	20.0	-	-	-	-	17,900 *	2530 *
GL400S-200	81.3	13.7	16.9	-	-	-	-	48,840 *	2380 *
GL400S-220	80.2	14.5	18.1	10 *	23 *	67 *	0.32 *	7060 *	1720 *
GL400M	78.0	81.8	104.9	50	31	19	0.30	8150	2360
GL400M-100	-	82.0	-	52	31	16	0.29	8660	2400
GL400M-120	-	81.7	-	-	-	-	-	10,830	2500
GL400M-140	-	85.6	-	-	-	-	-	22,640	2710
GL400M-150	-	81.3	-	-	-	-	-	21,770 *	2490
GL400M-160	79.4	81.8	103.1	48	32	20	0.27	10,090 *	2000 *
GL400M-180	79.1	79.9	101.0	-	-	-	-	4260 *	1470 *
GL400M-200	78.9	71.9	91.2	-	-	-	-	2840 *	1210 *
GL400M-220	79.9	51.8	64.9	7 *	47 *	46 *	0.62 *	2110 *	1040 *
GL400L	75.1	168.7	224.7	52	36	12	0.35	13,630	2590
GL400L-100	-	167.2	-	53	35	11	0.33	17,170	2680
GL400L-120	-	161.8	-	-	-	-	-	16,180 *	2380 *
GL400L-140	-	159.4	-	-	-	-	-	7090 *	1910 *
GL400L-150	-	148.6	-	-	-	-	-	5730 *	1760 *
GL400L-160	76.6	156.1	203.7	48	40	12	0.29	4930 *	1630 *
GL400L-180	77.3	138.0	178.5	-	-	-	-	2920 *	1240 *
GL400L-200	77.8	109.4	140.5	-	-	-	-	2260 *	1080 *
GL400L-220	79.3	91.9	115.8	20 *	60 *	20 *	0.89 *	1840 *	970 *

*^a^* GL400 samples prepared by PEG400 solvolysis of JC wood meal with various particle size distribution (JC-S < JC-M < JC-L) were subjected to heat treatment at temperatures of 100 to 220 °C for 1 h. *^b^* Yield of guaiacyl-type trithioethylphenylpropanes. *^c^* Based on relative contour integration ratio in HSQC NMR (Figure 3). *^d^* Determined by SEC using polyethylene glycol/poly(ethylene oxide) standards. * Identified only soluble heat-treated GL400 fractions for the respective analysis. -, not determined.

**Table 2:** ATR-FTIR spectral data for GL400L, heat-treated GL400L-200 and GL400L-220.

**GL400L**		**Gl400L-200**				**GL400L-220**			
		***^a^* Soluble and Insoluble**		***^b^* Insoluble Fraction**		***^a^* Soluble and Insoluble**		***^b^* Insoluble Fraction**	
**λ^−1^/cm^−1^**	***^c^* RA**	**λ^−1^/cm^−1^**	***^c^* RA**	**λ^−1^/cm^−1^**	***^c^* RA**	**λ^−1^/cm^−1^**	***^c^* RA**	**λ^−1^/cm^−1^**	***^c^* RA**
*^d^* 947	0.265	949	0.270	949	0.223	949	0.271	951	0.245
*^e^* 1350	0.483	1350	0.484	1354	0.628	1350	0.487	1356	0.645
*^f^* 1423	0.521	1425	0.534	1421	0.673	1427	0.542	1421	0.735
*^g^* 1462	0.730	1462	0.746	1460	0.890	1462	0.756	1460	0.952
*^h^* 1597	0.448	1597	0.465	1593	0.702	1597	0.470	1593	0.709

*^a^* heat-treated GL400 sample, *^b^* determined insoluble GL400L fractions for treatment temperatures of 200 and 220 °C, which did not dissolve in the solvent (2-propanol:0.2M NaOH, *v*/*v*:1/1) used for UV lignin analysis, *^c^* relative absorbance (A/A_1512_), *^d^* CH_2_ rocking vibration (PEG), *^e^* CH_2_ wagging vibration (PEG), *^f^* aromatic skeletal vibration with C–H deformation, *^g^* C–H bending of CH_3_ and CH_2_ groups, *^h^* aromatic skeletal vibration and C=O stretching.

**Table 3:** ATR-FTIR spectral data for GL400M, heat-treated GL400M-200 and GL400M-220.

**GL400M**		**Gl400M-200**				**GL400M-220**			
		***^a^* Soluble and Insoluble**		***^b^* Insoluble Fraction**		***^a^* Soluble and Insoluble**		***^b^* Insoluble Fraction**	
**λ^−1^/cm^−1^**	***^c^* RA**	**λ^−1^/cm^−1^**	***^c^* RA**	**λ^−1^/cm^−1^**	***^c^* RA**	**λ^−1^/cm^−1^**	***^c^* RA**	**λ^−1^/cm^−1^**	***^c^* RA**
*^d^* 949	0.223	949	0.258	949	0.244	949	0.280	949	0.240
*^e^* 1350	0.472	1350	0.484	1354	0.597	1350	0.494	1354	0.636
*^f^* 1427	0.508	1427	0.540	1423	0.645	1427	0.559	1421	0.684
*^g^* 1462	0.725	1462	0.756	1460	0.874	1462	0.769	1460	0.919
*^h^* 1597	0.431	1597	0.460	1593	0.679	1597	0.468	1593	0.709

*^a^* heat-treated GL400 sample, *^b^* determined insoluble GL400M fractions for treatment temperatures of 200 and 220 °C, which did not dissolve in the solvent (2-propanol:0.2M NaOH, *v*/*v*:1/1) used for UV lignin analysis, *^c^* relative absorbance (A/A_1512_), *^d^* CH_2_ rocking vibration (PEG), *^e^* CH_2_ wagging vibration (PEG), *^f^* aromatic skeletal vibration with C–H deformation, *^g^* C–H bending of CH_3_ and CH_2_ groups, *^h^* aromatic skeletal vibration and C=O stretching.

## Supplementary Materials

The following are available online, Figure S1: SEC molecular weight distribution profiles of the heat-treated GL400S (a), GL400M (b), and GL400L series (c). The samples were subjected to heat treatment at temperatures of 100–220 °C for 1 h.
