# Supplementary material for: Effect of Heat Treatment on the Chemical Structure and Thermal Properties of Softwood-Derived Glycol Lignin
Source: Molecules. 2020 Mar 5;25(5):1167. doi: 10.3390/molecules25051167 (PMC7179094; doi:10.3390/molecules25051167)
Supplement: Supplementary file 1 [file molecules-25-01167-s001.pdf]

Supplementary Materials

Effect of Heat Treatment on the Chemical Structure and Thermal Properties of Softwood-Derived Glycol Lignin

Thi Thi Nge[1*], Yuki Tobimatsu[2], Masaomi Yamamura[2], Shiho Takahashi[1], Eri Takata[1], Toshiaki Umezawa[2,3] and Tatsuhiko Yamada[1*]

[1]Center for Advanced Materials, Forestry and Forest Products Research Institute (FFPRI), 1 Matsunosato, Tsukuba, Ibaraki 305-8687, Japan

[2]Research Institute for Sustainable Humanosphere, Kyoto University, Gokasho, Uji, Kyoto 611-0011, Japan

[3]Research Unit for Development and Global Sustainability, Kyoto University, Gokasho, Uji. Kyoto 611-0011, Japan;

*Corresponding authors: thithi@affrc.go.jp (T.T.N.); yamadat@affrc.go.jp.(T.Y.)

List of Contents

Figure S1. SEC molecular weight distribution profiles of the heat-treated GL400S (a), GL400M, and GL400L series (c). The samples were subjected to heat treatment at temperatures of 100–220°C for 1 h.

Number of pages: 2

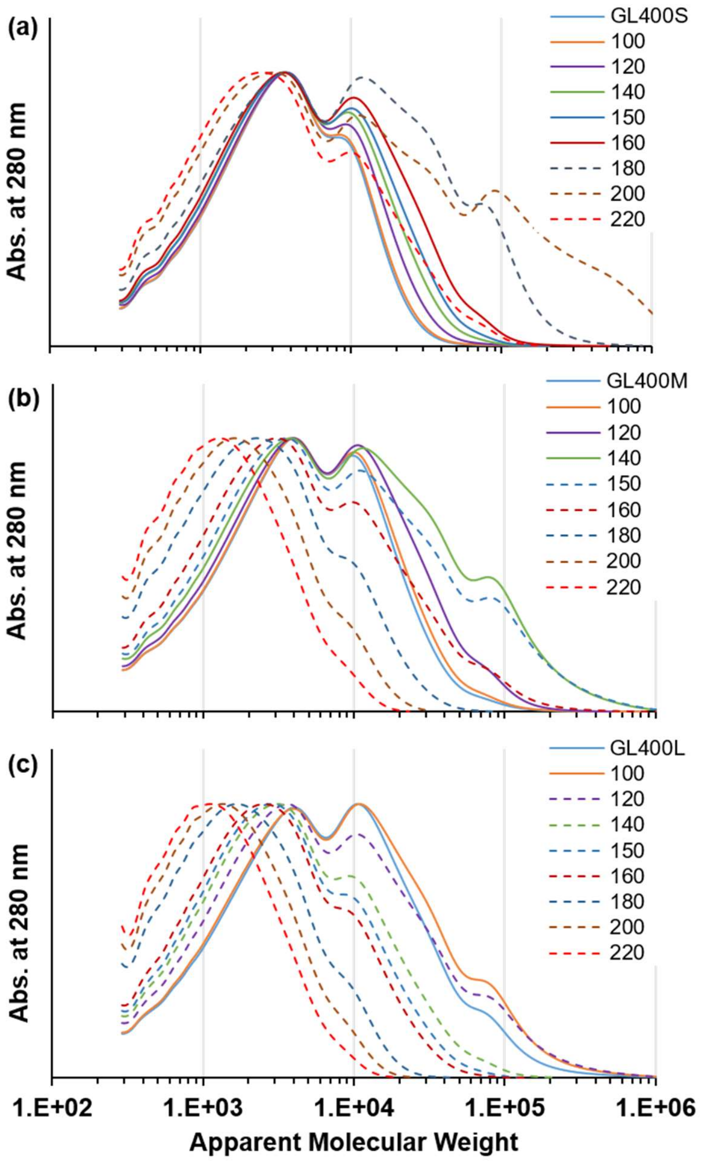

Figure S1. SEC molecular weight distribution profiles of the heat-treated GL400S (a), GL400M (b), and GL400L series (c). The samples were subjected to heat treatment at temperatures of 100–220°C for 1 h. The dot lines represented the soluble portion (10 mM LiBr/DMF) of heat-treated samples at corresponding heat treatment temperatures. GL400S, GL400M, and GL400L samples were prepared by PEG400 solvolysis of Japanese cedar (JC) wood meal with various particle size distribution of JC-S, JC-M, and JC-L (JC-S < JC-M < JC-L), respectively.
